# The antimalarial ferroquine: from bench to clinic

**DOI:** 10.1051/parasite/2011183207

**Published:** 2011-08-15

**Authors:** C. Biot, F. Nosten, L. Fraisse, D. Ter-Minassian, J. Khalife, D. Dive

**Affiliations:** 1 Unité de Catalyse et Chimie du Solide, CNRS UMR 8181, Université Lille Nord de France, Université Lille 1 BP 90108 59652 Villeneuve d’Ascq Cedex France Present address: Unité de Glycobiologie Structurale et Fonctionnelle, CNRS UMR 8576, IFR 147, Université Lille Nord de France, Université de Lille 1 59650 Villeneuve d’Ascq Cedex France; 2 Shoklo Malaria Research Unit, PO Box 46 Mae Sot Tak 63110, Thailand. Faculty of Tropical Medicine, Mahidol University, Bangkok 10400, Thailand. Centre for Clinical Vaccinology and Tropical Medicine, Nuffield Department of Clinical Medicine, University of Oxford, Oxford United Kingdom; 3 Sanofi-Aventis, Centre de Toulouse 195, route d’Espagne BP 13669 31036 Toulouse France; 4 Sanofi-Aventis Research and Development 1, avenue Pierre Brossolette 91385 Chilly Mazarin Cedex France; 5 CIIL, Inserm U1019, CNRS UMR 8024, Université Lille Nord de France, Institut Pasteur de Lille 1, rue du Pr Calmette 59019 Lille Cedex France

**Keywords:** malaria, bioorganometallics, drug candidate, ferroquine, mechanism of action, resistance, paludisme, bio-organométallique, candidat médicament, ferroquine, mécanisme d’action, résistance

## Abstract

Ferroquine (FQ, SSR97193) is currently the most advanced organometallic drug candidate and about to complete phase II clinical trials as a treatment for uncomplicated malaria. This ferrocenecontaining compound is active against both chloroquine-susceptible and chloroquine-resistant *Plasmodium falciparum* and *P. vivax* strains and/or isolates. This article focuses on the discovery of FQ, its antimalarial activity, the hypothesis of its mode of action, the current absence of resistance *in vitro* and recent clinical trials.

## Abstract (fr)

La ferroquine (FQ, SSR97193) est actuellement le candidat médicament organométallique le plus avancé dans son développement industriel et, en tant que traitement du paludisme non compliqué, il devrait bientôt voir s’achever des études cliniques de phase II. Ce composé à base de ferrocène est extrêmement actif contre des isolats et/ou des clones de *Plasmodium falciparum* et de *P. vivax* sensibles ou résistants à la chloroquine. Cet article présente la découverte de la FQ, son activité antipaludique, les hypothèses liées à son mode d’action, l’absence actuelle de résistance *in vitro* et, enfin, les récents essais cliniques.

## The Malaria Problem

With approximately 243 million cases and more than 800,000 deaths reported in 2009, malaria remains the most important human parasitic disease. Among the five *Plasmodium* species able to infect human, *P. falciparum* is responsible for most cases of severe disease and death, mainly in African children below the age of five. The morbidity caused by *P. vivax* in tropical countries outside of Africa has long been underestimated (Anstey *et al.*, 2009, Baird, 2009). Malaria is a factor of poverty in endemic countries (Stratton *et al.*, 2008). In the absence of an effective vaccine and reliable approaches for vector control, chemotherapy remains the corner stone of malaria control. Quinine has been the first widely used antimalarial drug. Synthetic derivatives of quinine were the 8-aminoquinoline primaquine and the 4-aminoquinoline chloroquine (CQ). When resistance to CQ emerged in the late 1950 s, the strategy was to modify the chemical structure of the existing compounds. The synthesis of CQ-like drugs led to the discovery of amodiaquine (AQ) and later mefloquine (MQ), halofantrine in the United States and lumefantrine in China (Baird, 2005). But the pace of new drug development has been slow and no new antimalarial drugs have been introduced into clinical practice since artemether-lumefantrine registered in 1998 (Olliaro & Wells, 2009). For all new antimalarial drugs introduced the risk of resistance can be reduced by combination therapy (White, 1999; Nosten & White, 2007). In 2006, the WHO guidelines recommended new treatments combining two drugs with different mechanisms of action. Treatments containing an artemisinin derivative (artemisinin-combination therapies, ACTs) are now standard treatment for *falciparum* malaria. However, a decline of susceptibility to artesunate has been recently reported in the Thai- Cambodian border region (Dondorp *et al.*, 2010). So the search for new molecules with antimalarial activity is more important than ever.

Many strategies can be used for the search of affordable and efficient antimalarial drugs. These strategies include ethnopharmacology (*i.e.* bio-evaluation of the efficiency of traditional medicines), medicinal chemistry, combinatorial chemistry and chemical libraries screening by high throughput screening, and drug design. These strategies have led to the discovery of potential antimalarials such as the synthetic endoperoxides and others (Dhanawat *et al.*, 2009). But the clinical development of new compounds is often been stopped for various reasons: toxicity, chemistry, pharmacology, or economics, and less than one in ten promising molecules that have entered the pipeline reaches the stage of clinical studies. In the mid- 90 s, we extended the strategy developed by Gérard Jaouen (Vessieres *et al.*, 1988) in anticancer therapy to antimalarial therapy (see Chavain & Biot, 2010 for review). The main antimalarials in current use (CQ, quinine, mefloquine, artemisinin, atovaquone) were modified by introduction of a ferrocenyl moiety in their chemical structure. More than 150 ferrocenic analogues have been synthesized, by us and others (Biot & Dive, 2010). The ferrocenic analogues were systematically tested against *in vitro* cultures of *P. falciparum* with CQ-susceptible and CQ-resistant strains. Ferroquine (FQ, SSR97193) was rapidly identified as a lead compound to meet candidate nomination requirements (Biot *et al.*, 1997). The clinical phase IIb study (efficacy/safety in adults, adolescents and children) began in 2009 in Africa.

This mini-review will focus on the discovery of FQ, its antimalarial activity, the hypothesis of its modes of action and recent clinical trials.

## The Organometallic Antimalarial Compound Set

**Fig 1.**
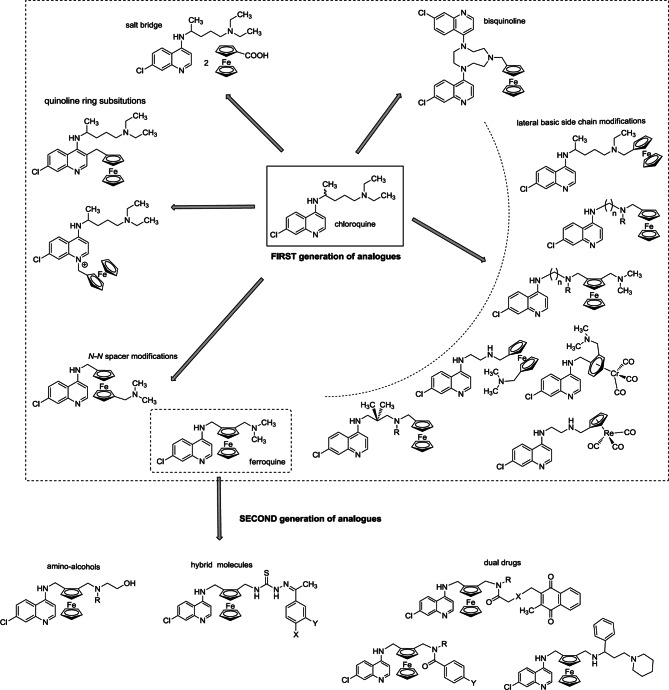
Scheme of different strategies adopted in synthesis of ferrocene-CQ hybrids.

Since 1993, we and others have systematically prepared organometallic versions of the antimalarials in current use such as CQ, primaquine, mepacrine, mefloquine, quinine, artemisinin, and atovaquone (see Dive & Biot, 2008 for review). New sandwiches and half-sandwiches metal complexes (Dunitz *et al.*, 1956) have been synthesized and characterized. *In vitro* tests of their antimalarial activity were performed. Other organometallic compounds with *a priori* unknown antimalarial activity were still screened. A collection of almost 150 compounds was made available. Among the organometallicdrug hybrids, the most interesting compounds were the ferrocene-drug hybrids and among those the ferrocene-chloroquine hybrids were the most promissing (Fig. 1).

Note here that the ferrocene-artemisitene hybrids showed also interesting properties with activities equal to artemisinin (Delhaes *et al.*, 2000, Dive & Biot, 2008). In the ferrocene-CQ hybrids series, we have shown that the ferrocene moiety has to be covalently flanked by a 4-aminoquinoline and an alkylamine (Biot *et al.*, 2006). Ferroquine (FQ, SSR97193) was the first compound synthesized by us (Biot *et al.*, 1997). Later, a second generation of analogues of FQ was designed and investigated. For example, we synthesized dual molecules including a FQ analogue conjugated with a glutathione reductase inhibitor or a glutathione depletory (Chavain *et al.*, 2009) Nevertheless, this strategy failed to identify a “new” lead for a further development. More interestingly, amino-alcohols based on the FQ structure are active against CQ-susceptible (CQS) and CQ-resistant (CQR) clones of *P. falciparum*. In addition, in this second generation of analogues the ferrocenic amino-alcohols exert antiviral effects with some selectivity toward SARS-CoV infection (Biot *et al.*, 2006b).

## Antimalarial Activity Of Ferroquine

### Antimalarial activity on laboratory clones

FQ antimalarial activity was compared to that of CQ with standard *in vitro* parasite growth inhibition method, based on tritiated hypoxanthin incorporation in erythrocytes infected with *P. falciparum*, incubated 48 hours (Desjardins *et al.*, 1978). Preliminary studies have shown that FQ was equally active as a base, ditartrate or dichlorhydrate salts (unpublished results).

**Fig 2.**
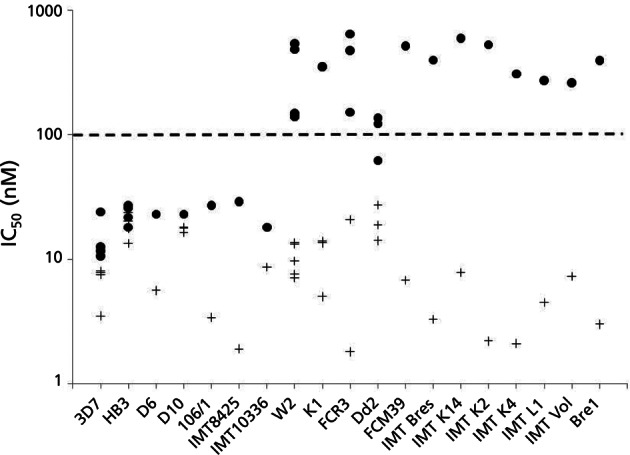
Susceptibility of 19 laboratory *P. falciparum* clones to CQ and FQ compiled from 11 different published studies. IC_50_ for CQ for each clone tested (l). + IC_50_ for FQ for each clone tested (**+**). The doted line indicate the threshold of resistance to CQ (Le Bras & Ringwald, 1990). References associated to each clone tested: 3D7 (1, 6, 8, 9, 10); HB3 (1, 7, 9, 10); D10 (2, 3, 4, 5); W2 (1, 6, 8, 9, 10); K1 (2, 3, 4, 5); FCR3 (1, 6, 11); Dd2 (7, 10, 11); D6, 106/1, IMT8425, IMT10336, FCM39, IMT Bres, IMT K14, IMT K2, IMT K4, IMT L1, IMT Vol, Bre1 (1). References: 1: Henry *et al.*, 2008; 2: Beagley *et al.*, 2002; 3: Beagley *et al.*, 2003, 4: Blackie *et al.*, 2007; 5: Blackie & Chibale 2008; 6 Biot *et al.*, 2006b; 7: Biot *et al.*, 1999; 8: Biot *et al.*, 2006a; 9: Daher, *et al.*, 2006a; 10: Daher, *et al.*, 2006b; 11: Delhaes *et al.*, 2001.

Tests results available from 11 studies performed in different laboratories and using 19 CQ^S^ and CQ^R^ *P. falciparum* laboratory adapted clones are represented in Fig. 2. The results show that the response to CQ can be easily dissociated between susceptible and resistant clones, which are spread respectively on either sides of the 100 nM IC_50_ for CQ. However, FQ is equally active on both types of clone and is at least equally active and often more active than CQ on CQ^S^ parasites. No resistance to FQ occurred in CQ^R^ clones and no correlation was found between susceptibility to FQ and polymorphism in transport proteins implicated in quinoline resistance (Henry *et al.*, 2008).

### *In vivo* antimalarial activity in rodent models

Antimalarial activity of FQ was tested on various rodent malaria strains (*P. berghei*, *P. yoelii*, *P. vinckei*) by the standard four day test of Peters (1987) adapted to determine the curative dose. On *P. berghei* N and *P. yoelii* NS strains, FQ and CQ had a close EC50 (treatment with a decrease in parasitaemia of 50% at the end of assay) and the simple four days test could not lead to conclude to a better efficacy of FQ *versus* CQ. But the curative tests are more significant and showed that *P. berghei* and *P. vinckei* infections were cured in presence of 8.3 mg/kg/d of FQ for four days when with CQ 30 to 55 mg/kg/d were necessary to cure CQ^S^ strains and the drug was unable to cure resistant strains, even at a toxic dose (Biot *et al.*, 1997, Delhaes *et al.*, 2001, Dive & Biot, 2008, Biot & Dive, 2010). Moreover, it has been shown that FQ was active not only by subcutaneous administration, but also by oral route, which was an interesting indication concerning the bioavailability of the drug by digestive tract. This was further confirmed by additional pharmacokinetic studies (Biot & Dive, 2010).

### Enantiomers

As FQ is a racemic compound. The two stereoisomers were synthetized and showed an antimalarial activity similar to that of the parent compound *in vitro* (Delhaes *et al.*, 2002).

### Metabolization and activity of metabolites

It was first postulated that the metabolism of FQ may share a common pathway with that of CQ and potential metabolites (*N*-monodemethyl-FQ and *N*didemethyl- FQ) were synthesized and tested (Biot *et al.*, 1999). The metabolism of FQ was then studied in details *in vitro* and enabled to determine its degradation pathway (Daher, *et al.*, 2006a). *In vitro* FQ is mainly metabolized to a major *N*-monodemethylated metabolite, SSR97213 (EVT0233) and to a further potential metabolite that is an *N*-didemethylated compound. Antimalarial activity of *N*-monodemethyl-FQ was found to be comparable to that of parent compounds on two CQ^S^ clones and remained much more active than CQ on two CQ^R^ clones. On the another hand, *N*-didemethyl-FQ had a decreased activity on CQ^R^ clones, mainly if IC_90_ of compounds is taken into account (Daher, *et al.*, 2006a).

### Efficacy on clinical isolates

Compounds were evaluated with standard *in vitro* parasite growth inhibition methods, in erythrocytes infected with *P. falciparum*, incubated at least 24 hours with the drugs. The antimalarial activity of FQ (SSR97193) on blood clinical isolates (CQ^S^, CQ^R^, and multi-drug resistant isolates) infected by *P. falciparum* was assessed in seven different studies of African patients (Senegal, Gabon) (Pradines *et al.*, 2001 & 2002; Atteke *et al.*, 2003; Kreidenweiss *et al.*, 2006), or southeast Asian patients (Chim *et al.*, 2004; Barends *et al.*, 2007) in comparison with existing antimalarial drugs. Data on FQ, CQ, and artesunate are reported in Table I.

**Table I.** Effect of FQ (SSR97193 – IC_50_ and 95% confidence intervals) on *P. falciparum* clinical isolates from different studies.

	Ferroquine			Chloroquine				Artesunate			
Country	n	IC_50_ (nM) *(ng/mL)*	95% CI	N	IC_50_ (nM) *(ng/mL)*	95% CI	% resistance	n	IC_50_ (nM) *(ng/mL)*	95% CI		Reference
Gabon	103	10.8	8.6-13.5	102	370	319-429	95 (a)	65	2.9	2.3-3.7		Biot et al., 1999
		*4.7*	*3 . 8-5.9*		*118.4*	*102-137*			*1.1*	*0.9-1.4*
Senegal	55	7.9	6.5-9.7	53	102	74-140	55 (a)	51	1.9	1.5-2.3		Biot et al., 2006a
		*3.4*	*2.8-4.2*		*32.6*	*23.7-44.8*			*0.7*	*0.6-0.9*
Gabon	56	16	14.4-17.8	56	141	70-285	52 (a)	nt	nt	nt		Daher, *et al.*, 2006a
		*6.9*	*6.3-7.8*		*45.1*	*22.4-91.2*				
Gabon	60	27.9	2.3-33.2	60	398	166-956	97 (a)	nt	nt	nt		Daher, *et al.*, 2006a
		*12.1*	*1.0-14.5*		*127.3*	*53.1-306*				
Thailand	65	9.3	8.7-10.0	62	341	304-382	100 (a)	56	4.0	3.1-6.3		Daher, *et al.*, 2006b
		*4.0*	*3.8-4.4*		*109.1*	*87.2-122*			*1.5*	*1.2-2 .4*
Gabon	40	1.9	0.6-6.7	43	113	12.4-332	100 (b)	43	1.0	0.2-6.0		Delhaes *et al.*, 2001
		*0.8*	*0.3-3.0*		*36.1*	*4.0-106*			*0.4*	*0.1-2.3*
Cambodia	155	29	26.3-31.6	155	135	121-151	32 (a)	150	1.1	1.0-1.2		EVT0231
		*12 6*	*11.6-13.8*		*43.1*	*38.7-48.2*			*0.4*	*0.4-0.5*

n = number of clinical sites; nt = not tested; CI = confidence interval; IC_50_ = inhibitory concentration decreasing a response by 50%; (a) = % of resistance using the threshold level of resistance IC_50_ > 100 nM; (b) = % of resistance using the threshold of IC_99_ > 30 nM for the HRP2 detection assay. For values in *italics*, units = ng/mL, calculated for translation to a free-base or free-acid gravimetric concentration.

Taking all these studies together, FQ was evaluated on 534 clinical isolates, 220 from Southeast Asia and 314 from Africa. In all these studies, FQ, like artesunate, displayed a very potent antimalarial activity against *P. falciparum* (range IC_50_ below 30 nM [13 ng/mL] for FQ and below 4 nM [1.5 ng/mL] for artesunate) with equal efficacy upon CQ^S^ and CQ^R^ clinical isolates (resistant isolates, with IC_50_ over 100 nM, represented from 32% to 100% of samples).

In addition, in the study from Thailand the main FQ *in vivo* metabolite (SSR97213) was investigated (Barends *et al.*, 2007). SSR97213 was shown to be highly potent against *P. falciparum* (N = 64, IC_50_ = 37 nM with 95% confidence intervals [CIs] = 34.3 to 39.9 nM, or IC_50_ = 16.0 ng/mL with 95% CIs = 14.9 to 17.3 ng/mL) on all the clinical isolates. To investigate whether *P. vivax* was sensitive to FQ a study was conducted in northwestern Thailand on 63 isolates collected from October 2006 to April 2009 to examine the effects of FQ and its demethylated metabolite (SSR97213) on the ring stage and the schizont maturation by microscopy. All samples were collected from patients with acute *P. vivax* who had mono-species parasitaemia of > 100/500 white blood cells. FQ was found to have a potent *ex vivo* effect on *P. vivax* schizont maturation (median IC_50_ = 15 nM; 75% CIs = 12 to 20 nM, n = 52) with SSR97213 being less active (IC50 = 77 nM; 75% CIs = 14 to 205 nM), and no significant cross-sensitivity between FQ and other antimalarials was detected; consequently FQ may be a suitable replacement for chloroquine in the treatment of drug-resistant *P. vivax* malaria (Leimanis *et al.*, 2010). In the Gabonese study (Kreidenweiss *et al.*, 2006), IC_99_ s were reported in comparison with IC_50_ s (Kreidenweiss *et al.*, 2006). For artesunate and FQ, the IC_99_ s were 5.76 nM (95% CIs = 0.57 to 49.1 nM) or IC_50_ = 2.21 ng/mL (95% CIs = 0.22 to 18.9 ng/mL), and 5.75 nM (95% CIs = 1.10 to 56.9 nM) or IC_50_ = 2.50 ng/mL (95% CIs = 0.48 to 24.8 ng/mL). These values are close to the reported IC_50_ s, indicating a strong potency and the ability to efficiently kill all parasites present in the field isolates. Finally, the susceptibility of *P. falciparum* isolates from Madagascar (n = 21), Guyana (n = 65) and Cambodia (n = 62) to FQ was measured at the local Pasteur Institutes using the [3H]-hypoxanthine incorporation method. The mean IC_50_ (with minimum and maximum IC_50_ values), were 5.96 nM (0.2-43.2), 8.68 nM (3.05- 55.77) and 10.18 nM (2.53-43.43), respectively (Eric Legrand, personal communication).

In all studies, no cross-resistance was observed with CQ and other antimalarials, although weak occurrences could be attributed, in one study to fluctuations of initial inoculums used for test (Kreidenweiss *et al.*, 2006). This absence of cross-resistance is supported by molecular studies, which showed that there was no association between polymorphims of resistance of *pfcrt* gene, the main molecular marker for CQ, and FQ susceptibility in field isolates (Daher, *et al.*, 2006b). This last observation was then extended to other markers of quinoline resistance (Henry *et al.*, 2008) and to *pymdr* and *pycrt* genes of the rodent strain *P. yoelii* (Dive & Biot, 2008).

### Resistance acquisition under ferroquine pressure

An *in vitro* study on *P. falciparum* resistance acquisition under ferroquine pressure was performed on human red blood cells infected with the W2 clone. After two months of FQ pressure we were unable to obtain a viable resistant strain. During these experiments however, we observed very few parasites, which were unable to develop when transferred in drug-free medium (Daher, *et al.*, 2006b).

An attempt to obtain a rodent FQR strain starting from *P. yoelii* resulted in a phenotype that was not fixed genetically the resistance disappearing as soon as FQ pressure was removed. Moreover, the phenotype was emerging very slowly and was confined strictly to reticulocytes and easily cleared by the host (Dive & Biot, 2008).

These results clearly show that the fitness cost of FQ resistance is very high for the parasite and that it would be detrimental for them in competition with non-resistant clones.

## Modes of Action: Hypotheses

CQ is thought to act by interfering with the digestion of haemoglobin in the blood stages of the malaria life cycle. Even if CQ and FQ share some similarities in their activity, FQ clearly showed important and additional mechanisms of action when compared to CQ (Table II) (Biot *et al.*, 2005; Dubar *et al.*, 2011).

**Table II.** Comparative properties of chloroquine (CQ) and ferroquine (FQ).

Properties	CQ	FQ
Intramolecular hydrogen bond	Yes	Yes
Weak base properties (p*K*_a1_ and p*K*_a2_ values)	10.03 and 7.94	**8.19 and 6.99**
Neutral and protonated forms at vacuolar pH	One time	**Ten times**
Lipophilicity at pH 5.2	- 1.2	- 0.77
Lipophilicity at pH 7.4	0.85	**2.95**
Complex with hematin and stoichiometry	Yes (1:1)	Yes (1:1)
Interaction with monomeric hematin (log K)	Yes (5.52)	Yes (5.52)
Inhibition of β-hematin formation (IRS)	Yes	Yes
BHIA_50_	1.9	**0.78**
Production of hydroxyl radicals	No	**Yes**
Activity on CQ^R^ clones and isolates	No	**Yes**
Relation with specific molecular resistance markers	Yes	**No**

IRS = infrared spectroscopy. BHIA_50_ = 50% inhibitory concentration for β-hematin inhibition in equivalents of compounds to hemin (Biot *et al.*, 2005).

The weaker base properties of FQ compared to CQ combined with its higher lipophilicity at pH 7.4 and the peculiar conformation provided by the intra-molecular hydrogen bond present in non polar conditions result in a better potency for FQ to cross membranes and a higher accumulation in the digestive vacuole. At the pH in that organelle, the physicochemical properties of FQ evidenced a higher fraction of neutral and mono-protonated forms and suggested a more efficient inhibitory activity on hematin biocrystallization (Dubar *et al.*, 2011), which was verified *in vitro* in BHIA (β-Hematin Inhibition Assay). Moreover, preferential localization of FQ at the site of crystallization of hemozoin close to the membrane of acidic vacuole might induce two independent or concomitant behaviours: first FQ might inhibit the self assembly of the hemozoin crystal and second FQ might specifically generate reactive oxygen species (*per se*, or *via* destruction of the hemozoin crystal) and induce lipid peroxidation and alteration of digestive vacuole (Chavain *et al.*, 2008; Dubar *et al.*, 2011).

All these properties might explain why FQ is more active than CQ *in vitro* even in a susceptible *P. falciparum* clone. The *in vitro* assays emphasized the specific importance of the intra-molecular hydrogen bond in FQ. Indeed in our studies based on methyl- FQ (an analogue of FQ without the intra-molecular hydrogen bond due to the presence of a methyl group on the 4-amino group), we clearly showed that the presence of the intra-molecular hydrogen bond allows FQ to escape resistance mechanisms and avoid crossresistance with the current antimalarials (Biot *et al.*, 2009; Dubar *et al.*, 2011).

## Clinical Trials

A total of 335 subjects, or patients have been administered with FQ (SSR97193) as of June 28 2010. In seven completed Phase 1/2 studies, 173 males subjects/patients were part of two trials performed in healthy Caucasian subjects, four trials conducted in asymptomatic African patients infected with *P. falciparum*, and one Phase IIa dose-escalation safety and activity (including adult African patients with mono-infection with *P. falciparum* and parasitemia within the 100 to 200,000/μL limits). Ongoing phase IIb dose-range study accounting for 440 patients conducted across seven African countries is currently assessing in four groups the safety and efficacy of an association of FQ-at a three dose level- with artesunate and FQ alone in patients with mono infection with *P. falciparum*. The first and second cohort consisting of adult/adolescent patients and children > 20 kg has been completed. Other potential combinations and indications are under evaluation at the time of writing this review.

## Conclusions and Perspectives

In conclusion, FQ clinical trials will enable the definition of conditions of use of this new antimalarial drug, which appears to be well positioned in the pipeline. One remaining question is the cause of the potent activity of the drug, mainly towards CQ resistant parasites, and its relation with the structure of the molecule. Some clues (role of the hydrogen bond, role of redox activity, nature of the metal present in the metallocene moiety) are currently under examination to clarify the mechanisms of entry of FQ in the infected red blood cell, its site and mechanism of action and its relation with the transporters involved in resistance against different aminoquinolines, which appear ineffective to expel the molecule out of the parasite. On the clinical front, it remains to determine how this new drug will be best combined with a partner to limit the risk of resistance.
